# Perforated Closed-Loop Obstruction Secondary to Gallstone Ileus of the Transverse Colon: A Rare Entity

**DOI:** 10.1155/2015/691713

**Published:** 2015-02-05

**Authors:** S. P. Carr, F. T. MacNamara, K. M. Muhammed, E. Boyle, S. M. McHugh, P. Naughton, A. Leahy

**Affiliations:** ^1^Royal College of Surgeons in Ireland, Dublin 2, Ireland; ^2^Department of Surgery, Beaumont Hospital, Dublin 17, Ireland

## Abstract

*Introduction*. Gallstone ileus (GSI) of the colon is an extremely rare entity with potentially serious complications including perforation. *Case Presentation*. An 88-year-old man presented to the emergency department with abdominal pain and distension. Clinical exam revealed signs of peritonism. Computed tomography (CT) revealed GSI of the transverse colon with a closed-loop large bowel obstruction (LBO) and caecal perforation. The patient underwent emergency laparotomy. A right hemicolectomy was performed, the gallstone was removed, and a primary bowel anastomosis was undertaken. A Foley catheter was sutured into the residual gallbladder bed to create a controlled biliary fistula. The patient recovered well postoperatively with no complications. He was discharged home with the Foley catheter in situ. *Discussion*. Gallstone ileus is a difficult diagnosis both clinically and radiologically with only 50% of cases being diagnosed preoperatively. Most commonly it is associated with impaction at the ileocaecal valve and small bowel obstruction. Gallstone ileus should also be considered as a rare but potential cause of LBO. This is the first reported case of caecal perforation secondary to gallstone ileus of the transverse colon. Successful operative management consisted of a one-stage procedure with right hemicolectomy and formation of a controlled biliary fistula.

## 1. Introduction

Most common mechanical bowel obstruction due to a gallstone occurs at the ileocaecal valve due to the reduced diameter and reduced active peristalsis [1]. This has been reported as being associated with jejunal perforation [2, 3].

Colonic obstruction due to a gallstone occurs rarely [4, 5]. The obstructing stone needs to measure at least 5 cm in diameter or larger than 2.5 cm with concurrent bowel pathology [6–8]. Colonic perforation in this setting is rare with only three previous reported cases of sigmoid perforation. This is the first reported case of perforation secondary to gallstone ileus (GSI) of the transverse colon resulting in close-loop large bowel obstruction (LBO).

## 2. Case Report

An 88-year-old man presented to the Emergency Department in a tertiary Irish hospital with abdominal pain and distension having being referred by his general practitioner. His symptoms had occurred three days prior to presentation. There was no associated nausea or vomiting. The patient had no previous medical or surgical history and was on no regular medications. He had no previous known history of gallstones.

**Figure 1:**
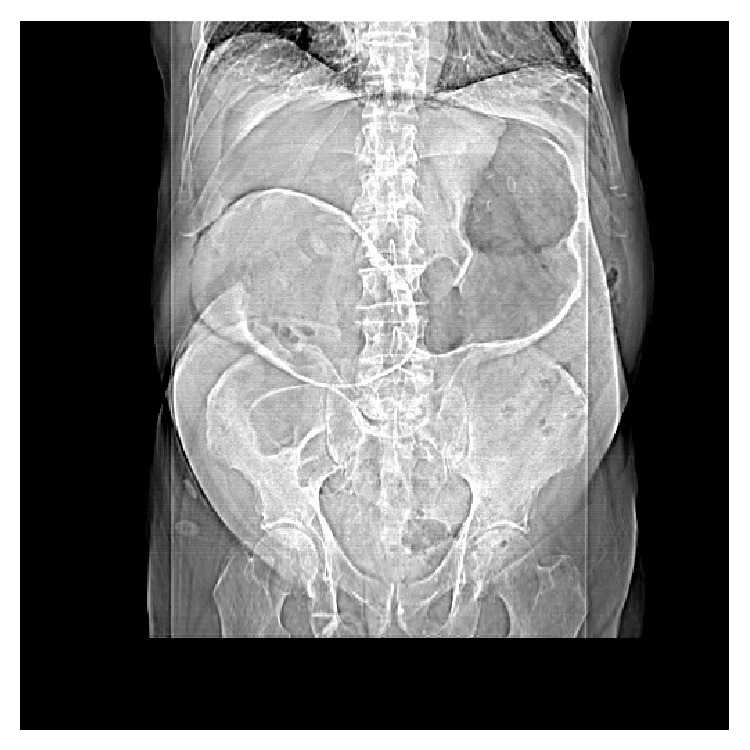
Abdominal X-ray revealing distended large bowel with a collapsed small bowel.

**Figure 2:**
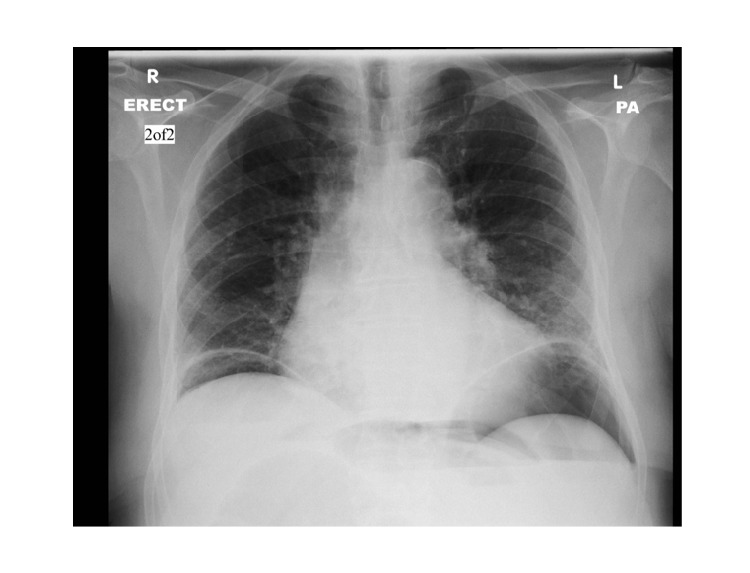
Chest X-ray revealing free intraperitoneal air under the diaphragm.

An abdominal X-ray and erect chest X-ray were requested. His abdominal X-ray revealed a distended large bowel with a collapsed small bowel (Figure 1). His chest X-ray revealed free intraperitoneal air visible under the diaphragm (Figure 2). Computed tomography (CT) scan of the abdomen and pelvis was performed. This revealed a close-loop LBO at the level of the transverse colon, with caecal distention to 10 centimetres, pneumatosis of the caecal wall, and free intraperitoneal air and fluid consistent with enteral perforation. The patient was subsequently scheduled for emergency laparotomy.

**Figure 3:**
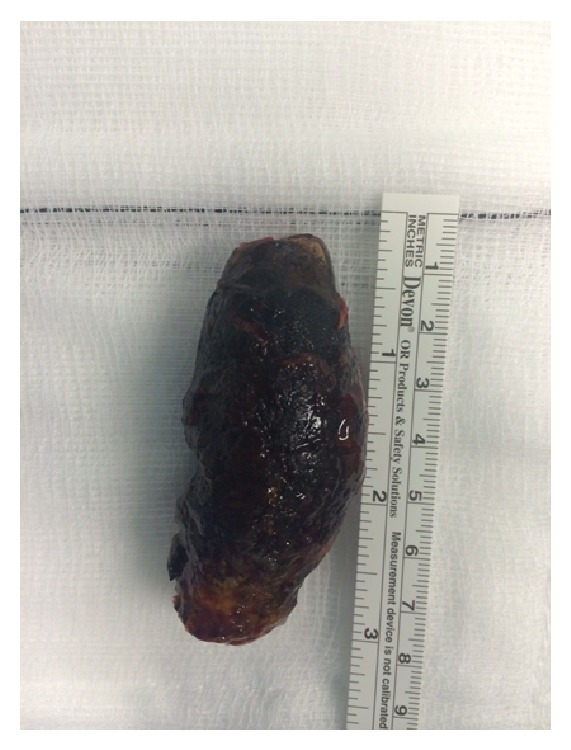
Photograph of the large 7 cm gallstone removed from the transverse colon.

**Figure 4:**
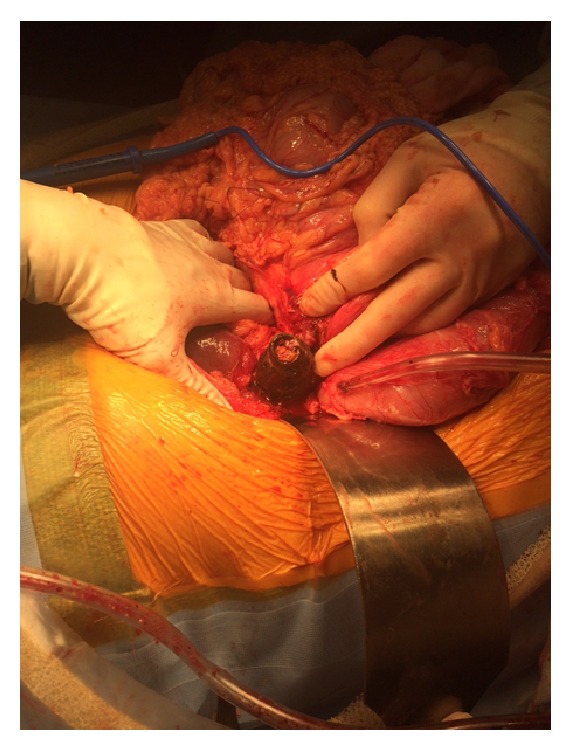
Intraoperative picture showing 7 cm gallstone causing obstruction at the proximal third of the transverse colon, with a cholecystocolic fistula communicating between the transverse colon and the gallbladder.

**Figure 5:**
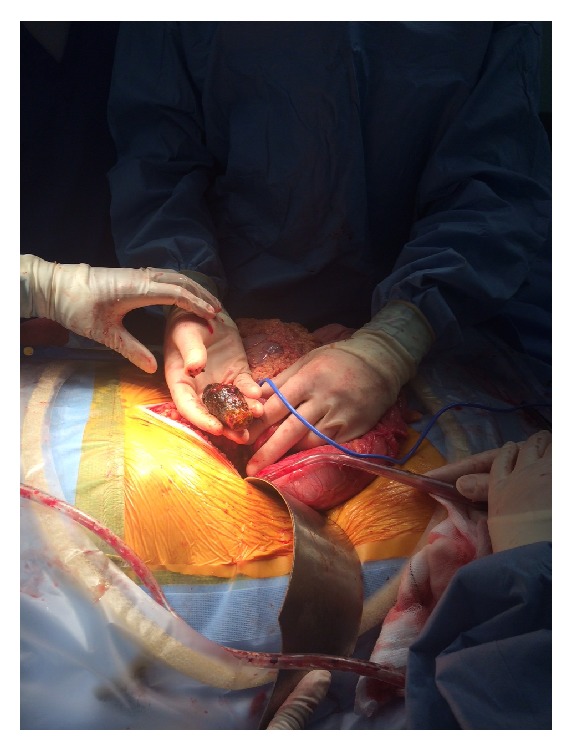
Intraoperative picture showing 7 cm gallstone being removed from the transverse colon.

The patient underwent a vertical midline laparotomy. The right colon was noted to be grossly distended with caecal ischemia and perforation. A seven-centimetre gallstone was noted to be causing obstruction at the proximal third of the transverse colon, where a cholecystocolic fistula communicating between the transverse colon and the gallbladder was identified (Figures 3, 4, and 5). The decision was taken to perform a right hemicolectomy that was performed from the terminal ileum to healthy transverse colon. A primary side-to-side stapled anastomosis was performed. Due to the degree of inflammation around the gallbladder and duodenum a complete cholecystectomy was not performed. A controlled fistula from the gallbladder bed to the skin was created via the insertion of a Foley catheter through a separate upper abdominal incision. Five millilitres of sterile water was inflated into the Foley catheter balloon, and a purse-string suture was used on the gallbladder wall around the catheter to hold the balloon in the gallbladder bed. A complete abdominal washout was performed and two Robinson drains were placed via separate incisions in the abdominal cavity.

Postoperatively the patient recovered well. The patient recommenced oral intake day three postoperatively and completed a ten-day course of intravenous antibiotics. He was discharged home with the Foley catheter in situ, where it was subsequently removed in surgical outpatients.

## 3. Discussion

It is acknowledged that GSI is a difficult diagnosis both clinically and radiologically with only 50% of cases being diagnosed preoperatively [9]. Only 50% of patients presenting with GSI have a history of gallstones or biliary pathology [10]. Considering the high mortality rate (15–18%) [1] associated with GSI, prompt diagnosis and appropriate surgical management are essential for a favorable outcome.

With regard to imaging techniques, CT is recognized as having the greatest diagnostic accuracy with a sensitivity of 93% and specificity of 100% [11]. In an emergency setting CT provides a more rapid and accurate diagnosis [12] and also allows for a better determination of the degree of obstruction, its location and visualisation of bilioenteric fistulae, and the condition of the adjacent bowel mucosa. Despite the number of modern radiological diagnostic modalities GSI involving the colon is such a rarity diagnosis is still most often made on laparotomy [13].

Debate exists regarding surgical options for GSI causing uncomplicated small bowel obstruction. Enterotomy alone with removal of the obstructing gallstone alone with expectant management is regarded as the most appropriate surgical management in an emergency setting [1, 9]. Single-stage surgery comprises a combination of enterolithotomy, cholecystectomy, and fistulotomy. Enterotomy alone is associated with a lower mortality rate (11.7%) when compared to single-stage surgery (16.9%). With regard to intraoperative fistula management, there is a low rate of further complications reported as a result of not performing a fistulotomy at primary surgery with only 5% recurrence rate of GSI. Furthermore, only 10% of patients require further surgery for persistent biliary symptoms [1]. In this case, however, in order to treat the caecal perforation a right colectomy was performed necessitating the operative treatment of the cholecystocolic fistula.

There is a paucity of reported cases involving colonic perforation. In 2009, van Kerschaver et al. reported on GSI causing necrosis and perforation of the rectosigmoid junction. A Hartmann's procedure was performed to treat the perforation and the obstruction. A subsequent cholecystectomy and closure of the cholecystocolonic fistula were planned to be performed at the time of restoration of the intestinal continuity [14]. D'Hondt and Schoofs also reported on the surgical management of sigmoid perforation secondary to GSI in 2011 and 2010, respectively [15, 16]. All of these previous cases report on GSI causing pressure-related perforation at the site of obstruction. Our case is unique in that we report the surgical management of a remote perforation of the caecum as a result of GSI causing LBO. Furthermore, octogenarians may have an incompetent ileocecal valve which would negate the evolution of a closed-loop obstruction. This was not the case in our reported patient. Successful patient outcome was achieved through prompt preoperative diagnosis and successful single-stage surgery involving right hemicolectomy and controlled biliary fistula formation.

## 4. Conclusion

Gallstone ileus causing a LBO is a rare entity, and cases involving colonic perforation are rarer still. Previous reported cases have demonstrated colonic perforation at the site of mechanical obstruction. We report on a closed-loop LBO leading to remote perforation secondary to GSI at the transverse colon. Successful management included right hemicolectomy and controlled biliary fistula formation. Gallstone ileus should be considered in patients as a potential cause of bowel obstruction, even without previously known history of gallstones.
